# Valorisation of Whey Permeate in Sequential Bioprocesses towards Value-Added Products–Optimisation of Biphasic and Classical Batch Cultures of *Kluyveromyces marxianus*

**DOI:** 10.3390/ijms24087560

**Published:** 2023-04-20

**Authors:** Karolina Drężek, Maria Krystyna Sobczyk, Zoltán Kállai, Anna Detman, Paula Bardadyn, Jolanta Mierzejewska

**Affiliations:** 1 Department of Drug and Cosmetics Biotechnology, Faculty of Chemistry, Warsaw University of Technology, 00-664 Warsaw, Poland; 2 Department of Genetics and Applied Microbiology, University of Debrecen, H-4032 Debrecen, Hungary; 3 Laboratory of White Biotechnology, Institute of Biochemistry and Biophysics, Polish Academy of Sciences, 02-106 Warsaw, Poland

**Keywords:** whey permeate, valorisation, *Kluyveromyces marxianus*, 2-phenylethanol, ethanol

## Abstract

Whey permeate is categorised as hazardous wastewater for aquatic environments, mainly due to its high lactose content. Therefore, it must be valorised before being released into the environment. One pathway for whey permeate management is its use in biotechnological processes. Herein, we present roads for whey permeate valorisation with the *K. marxianus* WUT240 strain. The established technology is based on two bioprocesses. During first, 2.5 g/L 2-phenylethanol and fermented plant oils enriched with different flavourings are obtained after 48 h biphasic cultures at 30 °C. The second process leads to a maximum of 75 g ethanol/L (Y_P/S_ = 0.53 g/g) after 96 h at 30 °C. Moreover, established whey permeate valorisation pathways reduced its biochemical oxygen demand and chemical oxygen demand values by 12- to 3-fold, respectively. Together, the present study reports a complete, effective, and environmentally friendly whey permeate management strategy while simultaneously enabling the acquisition of valuable compounds with substantial application potential.

## 1. Introduction

Biowaste management in a sustainable and environmentally-friendly manner is becoming a critical issue in protecting the ecosystem and for economic reasons. Currently, a growing trend toward the development of ‘green’ biotechnologies can be observed. Numerous resources, i.e., bio-compounds and biofuels, can be recovered from biowaste. For instance, in order to diversify energy sources, we move from fossil fuels to bioethanol. We treat sewage to save water and strongly emphasize waste conversion. In all these processes, microorganisms play an essential role, and particularly yeasts, are of great value.

Nowadays, all non-*Saccharomyces* yeasts are named by biotechnologists as ‘non-conventional’. Such yeasts include representatives of the *Candida*, *Yarrowia*, *Kluyveromyces*, *Pichia*, *Debaryomyces* or *Hansenula* species [1]. Non-conventional yeasts are considered valuable industrial hosts due to their preferential characteristics, namely thermos-, osmo-, and xerotolerance, salt resistance, and ability to metabolize diverse carbon sources or tolerate several by-products and inhibitors. Therefore, they are an excellent alternative for the industrial production of bioethanol, fine chemicals, lipids and recombinant proteins.

One of the most auspicious non-conventional yeast is *Kluyveromyces marxianus*. It occurs naturally in wide-ranging habitats such as fermented traditional dairy products, sugar industry sewage, plants, or sisal leaves. *K. marxianus* has GRAS (Generally Recognised as Safe) and QPS (Qualified Presumption of Safety) statuses. Representatives of this genus show high thermotolerance, with a significant growth efficiency of 0.99 1/h at 40 °C [2]. Moreover, the broad alternative substrate spectrum which can be metabolised (i.e., dairy industry waste such as cheese whey and its permeate, hemi-cellulose hydrolysates, sugar syrup molasses) is also one of *K. marxianus’* key characteristic traits. All these desired features make *K. marxianus* a versatile host for applications in the food, feed, and pharmaceutical industries [3,4,5]. To date, examples of *K. marxianus* usage include (1) extracellular proteins (e.g., β-galactosidase, β-xylosidase, inulinase, pectinase, carboxypeptidases); (2) single cell proteins (SCPs); (3) single cell oils (SCOs) and fatty acids; (4) aroma compounds (e.g., ethyl acetate, 2-phenylethanol, isoamyl alcohol); or (5) second generation bioethanol production [6]. Strains of this species were also used in terms of bio-environmental applications, helping to reduce organic loads of waste and remove toxic heavy metals or dyes from contaminated solutions [6,7]. An example of such raw material is whey permeate (WP, also known as dairy permeate). It is generated by the removal of proteins and other solids from whey resulting in a product with a high content of lactose, up to 80 g/L. Due to the above-mentioned composition, WP poses a significant risk to the environment when released directly into a watercourse. It is related to its organic load, manifested by high chemical and biochemical oxygen demand values (abbreviated as COD and BOD5, respectively). Year after year dairies produce increasing amounts of this waste and according to the reports, the WP market is estimated to acquire a size of over USD 970.6 million by the end of 2028 [8]. In view of the abovementioned facts, it is necessary to seek pathways for its development. 

Many dairies have been spray-drying WP and selling it for animal feed. Some parts are used in the food industry for various functional and nutritional benefits, such as the browning of baked goods and imparting a pleasant, caramelised flavour. There are also reports showing the possibility of using WP to produce some valuable molecules, e.g., acid whey permeate was described as an alternative growth medium for microalgae *Tetradesmus obliquus* and production of β-galactosidase [9]. With engineered *Escherichia coli*, Pasotti et al. [10] reported lactose to ethanol fermentation, reaching 40 g/L. Krische et al. [11], on the other hand, used whey permeate in the continuous culture of *Lactobacillus casei* subsp. *casei* and obtained L-lactic acid with a productivity of 10 g/L acid per hour. The abovementioned examples show that the biotechnological way can be an interesting alternative to managing WP while obtaining diverse compounds. Moreover, the lactose to selected product fermentation can complete the whey permeate valorisation chain by decreasing dairy waste polluting potential. Yet in order to utilize the lactose and the inorganic compounds present in WP, it is crucial to select an appropriate strain of the microorganism. The ideal candidate seems to be the lactose-fermenting yeast *K. marxianus*. 

In the present paper, novel ways for WP management with the use of the *K. marxianus* WUT240 strain have been proposed. The concept of the study is based on two bioprocesses. First, two-phase cultures, leading to two main products with potential use as cosmetic raw materials, i.e., 2-phenylethanol (2-PE, rose aroma with additional antimicrobial properties) and fermented plant oils (enriched, i.e., with flavouring substances and natural preservatives). Second, resulting in bioethanol that can be used as, e.g., disinfectant, or transportation fuel. Additionally, the two processes are coupled by the use of yeast biomass. The research concept was to develop the technology in such a way as to minimize the amount of waste generation while maximising the number of valuable products that can be obtained. The question was whether we would be able to efficiently produce the desired products and simultaneously reduce the environmental loads of WP examined in terms of chemical oxygen demand (COD) and biochemical oxygen demand (BOD5) values, thus confirming the possibility of neutralising this waste.

## 2. Results

The most used biotechnological route of producing 2-phenylethanol (2-PE) and its derivative 2-phenylethyl acetate (2-PEA) is a yeast-based bioconversion of L-phenylalanine (L-phe) via Ehrlich pathway [12]. In this process, L-phe is converted into phenylpyruvate, which is later decarboxylated to phenylacetaldehyde and then reduced to 2-PE through dehydrogenation. Thereafter, 2-PE can be transesterified to 2-PEA [13]. Therefore, different yeast strains are receiving interest in catalysing L-phe bioconversion to 2-PE for developing an efficient biotechnological production process [14,15]. 

**Figure 1:**
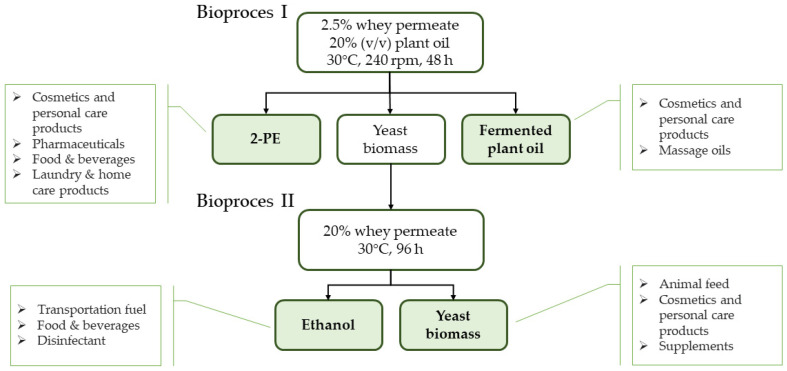
Conceptual diagram of the whey permeate valorisation process.

Research previously conducted by our team has shown that it is possible to increase the yield of 2-PE in a synthetic medium using a two-phase culture with the classic yeast *Saccharomyces cerevisiae* [16]. We also examined the chance of using the waste–whey permeate–to produce 2-PE in classic batch and continuous system with lactose-fermenting yeast *K. marxianus* WUT240 [17]. In this study, we wanted to explore the possibility of coupling these two technologies into a single process (schematic representation is depicted in Figure 1). This would enable not only the in situ removal of part of 2-PE from the culture and hence, prolong the metabolic activity of microbes (2-PE in a titer of 2–4 g/L is toxic for yeast [18]) but also obtain an additional interesting product, namely fermented plant oil enriched, i.e., in volatile organic compounds such as 2-PE and its derivatives. Moreover, yeast biomass multiplied in the first stage, which is typically considered a waste, was also examined if it could act as a biocatalyst in the subsequent process phase. The development of another WP-based microbial technology is likewise significant in reducing its harmful impact on the environment.

### 2.1. Whey Permeate Valorisation Enables 2-Phenylethanol and Fermented Plant Oils Production–Study on Bioprocess I

It is well known that fatty acids can be used as carriers of aromatic substances in a process known as ‘Enfleurage’ [19]. We have shown in our previous studies that rapeseed oil can be used in two-phase cultures with *S. cerevisiae* WUT3 strain [16]. Herein, we examined the possibility of using three plant oils (linseed, rapeseed, rice), selected based on their possible use in cosmetics, in cultures with *K. marxianus* WUT240.

The biocompatibility of the solvent is crucial in a whole-cell biocatalytic process. Therefore, in the beginning, the influence of selected oils on yeast growth was tested. Growth was monitored by OD600 (optical density at λ = 600 nm) in the aqueous phase of the biphasic systems. Organic solvents were added to the medium at an OD600 of 0.8–1.0 (the organic phase comprised 20% (*v*/*v*) of the overall culture, based on our previous estimates). 

**Figure 2:**
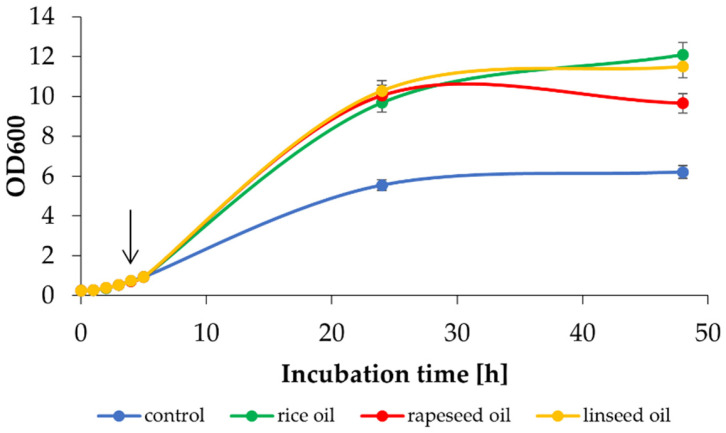
Effect of the presence of exogenous plant oils (20% (*v*/*v*)) on the growth of *K. marxianus* WUT240 in comparison with a control culture without addition of organic phase. Cells were grown in medium WP25 with an initial OD600 = 0.15. The second phase (rice oil, linseed oil or rapeseed oil) was added at OD600 of approx. 0.8–1.0 (indicated by an arrow).

As shown in Figure 2 the addition of oil to the cultures not only enables efficient yeast growth but also significantly improves it, in comparison to control (without oil addition). Up to a 95% increase in final OD600 value was noted with rice oil as a second phase (12.10 ± 0.15 vs. 6.21 ± 1.23). A lower, yet still substantial increase (approx. 55%), was obtained with rapeseed oil (9.66 ± 0.27 vs. 6.21 ± 1.23). Improved growth may be related to the ability to use carbon compounds present in oils as a source of energy.

After determining the lack of negative effect of oils on yeast growth, in parallel, we estimated the key parameters of 2-PE bioproduction by the *K. marxianus* WUT240 strain. The concentration of lactose, L-phe, and 2-PE in cultures was estimated by HPLC analysis after 48 h of incubation. In addition, at the end of the cultures, the phases were separated by centrifugation and the 2-PE was extracted from the oils with acetonitrile. Obtained results are summarised in Table 1. 

In all tested variants, lactose was utterly consumed after 48 h. At the same time, around 4 g/L of L-phe was metabolised, leading to 2.45 ± 0.17 g/L 2-PE in control cultures, and 1.7–1.8 g/L in aqueous and 4.1–4.4 g/L 2-PE in the organic phase of the two-phase system. The calculated product yield coefficient was 15% lower in the two-phase cultures compared to the control and 22% lower than the maximum coefficient value ($Y_{P/S}^{max}$= 0.75). Nevertheless, the results are satisfactory and prove the possibility of using whey permeate as a key component of the medium with *K. marxianus* WUT240. Furthermore, at the end of the cultures, not only pure 2-PE but also fermented plant oils enriched in 2-PE were obtained. 

**Figure 3:**
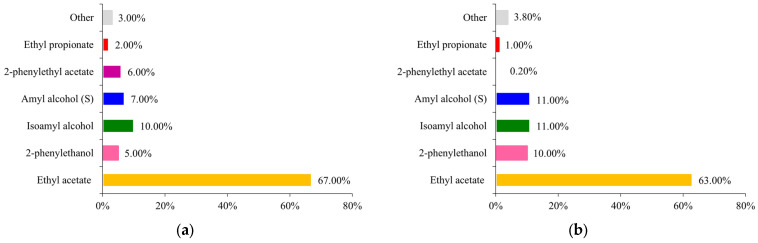

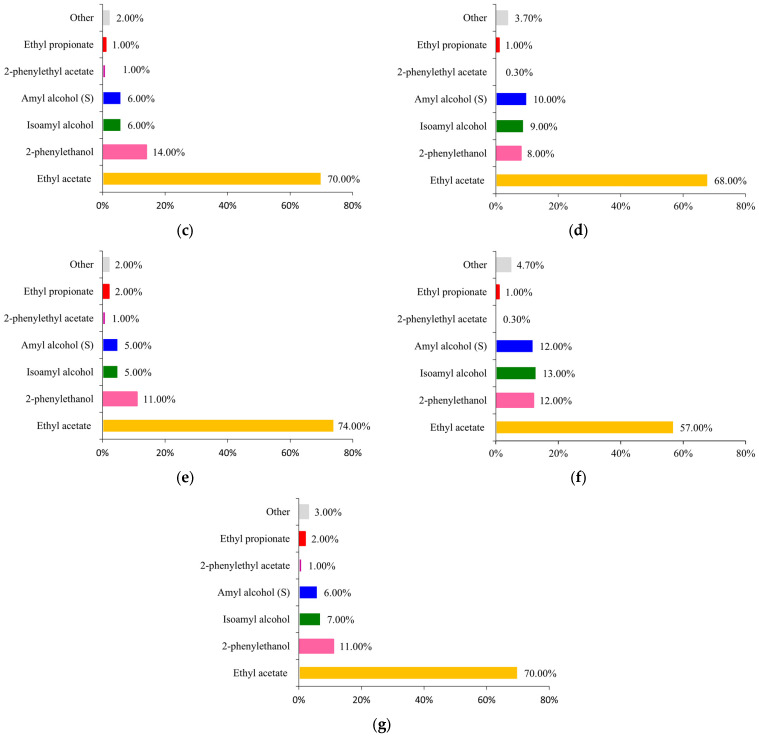
VOCs profile in aqueous and organic samples obtained in *K. marxianus* WUT240 cultures in WP25 medium incubated at 30 °C, 240 rpm for 48 h: (**a**) control culture; (**b**,**d**,**f**) aqueous phase profile in two-phase cultures with rice oil, rapeseed oil and linseed oil, respectively; (**c**,**e**,**g**) organic phase profile in two-phase cultures with rice oil, rapeseed oil and linseed oil, respectively.

*Kluyveromyces marxianus* strains can synthesise various aromatic compounds, such as alcohols, furanones, fruit esters, ketones, carboxylic acids, and aromatic hydrocarbons [20,21]. It has been previously reported that representatives of *Kluyveromyces* species can metabolize lactose and form ethyl acetate [22,23]. Thus, the question remained if any volatiles, other than 2-PE, were synthesised during two-phase cultures. Therefore, we determined selected volatile organic compounds (VOCs) in aqueous and organic phase samples using GC-MS (Figure 3).

The analysis of VOCs, expressed in the percentage of all identified substances, showed that the principal compound present in samples is ethyl acetate, comprising 57–68% in aqueous and around 70–74% in oil phases. 2-PE is around the second (for oil phases) or third (for aqueous phases) place and accounts for an average of 10% of all molecules detected. Significantly, its content more than doubled after the application of the two-phase system. Besides, as primary VOCs isoamyl alcohol (banana, rummy aroma), amyl alcohol (rummy aroma), 2-phenylethyl acetate (2-PE derivative of rose, floral aroma) and ethyl propionate (fruity, rummy aroma) were identified. A certain mixture of VOCs present in the oil phase not only gives it its characteristic sweet-fruity-floral aroma but also protects the resulting bioferments from contamination as the VOCs exhibit antimicrobial properties [24,25,26].

The level of VOCs produced seems to be dependent on the sugar metabolism in *K. marxianus* cells. During our previous studies on a synthetic medium, with glucose and sucrose as carbon sources, the qualitative VOCs profile was similar to the one presented here. However, a significant difference can be observed in terms of quantitative analysis (for comparison, see Supplementary Figure S1). On synthetic medium, higher 2-PE and its derivative 2-PEA content can be reached (up to 33% 2-PE and 11% 2-PEA of all VOCs). Additionally, around 15–28% lower ethyl acetate amount was noted. At least two explanations for this phenomenon are possible. According to the literature, lactose present in WP might favour ethyl acetate formation [27]. Additionally, the presence of other than L-phe nitrogen sources in WP can limit 2-PE bioconversion. It is commonly known that efficient biotransformation of L-phe to 2-PE can occur when the amino acid preferentially is the only nitrogen source in the medium [28]. 

### 2.2. Efficient Management of Whey Permeate with K. marxianus WUT240 Strain Is Possible in a Laboratory-Scale Bioreactor 

The next research task was to scale the process of whey permeate valorisation from batch flask cultures to a 4.8-L bioreactor. Because no significant difference was observed in variants tested on the Erlenmeyer flask scale, rice oil was chosen as a second phase. Two batches were conducted at 30 °C, with the airflow rate maintained at 0.8 vvm (gas volume flow per unit of liquid volume per minute) and different stirring speeds (250 or 500 rpm). During cultivation, the content of key compounds (lactose, L-phe, and 2-PE) was measured by HPLC. Obtained results are summarised in Table 2.

Since, to our best knowledge, there are no literature data on two-phase cultures with *Kluyveromyces marxianus* strains, the culture parameters were selected experimentally. In the first step, we scaled the volume of the culture 20 times, keeping the factors from the shaking flask experiments. This resulted in a reduction in the total yield of 2-PE production by as much as 41% (1.38 ± 0.03 g/L vs. 2.33 ± 0.04 g/L), even though yeast biomass almost doubled (12.51 ± 2.72 g DCW/L vs. 6.43 ± 0.53 g DCW/L). During the culture, 3.62 ± 0.45 g/L ethanol was also synthesised. As the major formation of inhibitory ethanol should be prevented, the two-phase system needs vigorous stirring and aeration. However, this can provoke the formation of a stable emulsion as the solvent will extract substances from the cell wall that act as emulsifiers [29]. Therefore, in the subsequent experiments, it was decided to increase the stirring speed by two times, from 250 rpm to 500 rpm, while maintaining the same airflow. This approach has resulted in a 27% rise in 2-PE production efficiency (2PE_tot_ increased from 1.38 ± 0.03 g/L to 1.90 ± 0.04 g/L). No ethanol formation was also noted. The results prove the process’s scalability, which is both important in obtaining metabolites of interest and managing significant amounts of whey permeate.

### 2.3. Use of K. marxianus WUT240 Biomass Enables Efficient Fermentation of Lactose to Ethanol in Cheese Whey Permeate–Study on Bioprocess II

**Figure 4:**
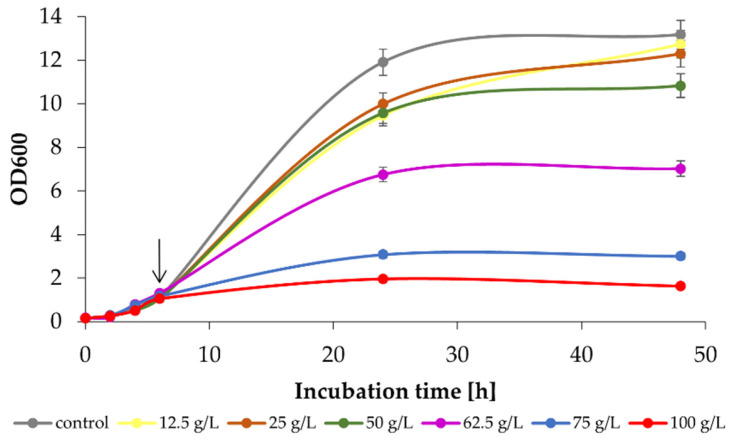
Effect of the presence of exogenous ethanol on the growth of *K. marxianus* WUT240 in comparison with a control culture without ethanol addition. Cells were grown in a SAB medium with an initial OD600 = 0.15. Ethanol at different final concentrations (12.5, 25, 50, 62.5, 75, and 100 g/L) was added when cultures reached OD600 of approx. 0.8–1.0 (indicated by an arrow).

During fermentation, yeast cells are exposed to unfavourable conditions. A high sugar concentration is needed to obtain high ethanol yields. However, because of such conditions, most yeast strains cannot grow and ferment high gravity substrate; osmotic stress leads to decreased growth and cell viability [30]. In this regard, it is crucial to determine the ‘safe’ range of substrate and product concentrations. Therefore, before target production cultures, the effect of exogenous ethanol on the growth of *K. marxianus* WUT240 was tested. Growth was monitored by OD600 measurements. Ethanol was added to the medium at an OD600 of 0.8–1.0 (the final concentration was between 12.5 and 100 g/L). As a control, a culture without ethanol addition was conducted. As shown in Figure 4, a minor reduction in the final OD600 value was measured for cultures containing 50 g/L ethanol (13.17 ± 0.20 vs. 10.82 ± 0.26). A more pronounced difference can be observed at 62.5 g/L ethanol–47% reduction in OD600 (7.01 ± 0.88 vs. 13.17 ± 0.20). Higher ethanol content almost entirely inhibited yeast growth, by 77% and 88% at 75 g/L and 100 g/L ethanol.

Simultaneously, the effect of lactose on yeast growth was also evaluated. For this purpose, 10-fold dilutions (up to 10^−7^) of overnight *K. marxianus* WUT240 cultures in SAB were dotted in four repetitions on plates containing rising lactose concentrations (up to 400 g/L). After 48 h incubation at 30 °C, yeast growth was monitored and described as log (cfu/mL).

**Figure 5:**
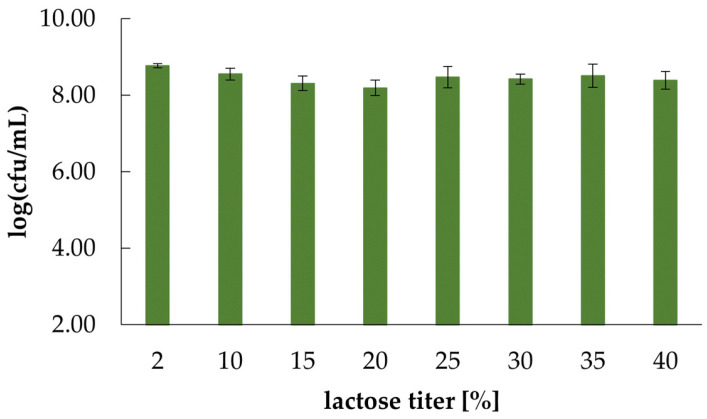
The results of the tolerance test for increasing concentrations of lactose (2–40% (*w*/*v*)). Cells were grown overnight in a SAB medium and thereafter serially diluted to 10^−7^. 10 µL samples of 10^0^–10^−7^ dilutions were spread on YNB-lactose medium in four repetitions and incubated at 30 °C for 48 h. Colonies were examined, and cfu/mL was calculated according to Equation (9).

The solubility of lactose in water is temperature dependent; e.g., 18.90 g at 25 °C, 25.15 g at 40 °C, and 37.21 g at 60 °C per 100 g solution can be dissolved [31]. During the preparation of plates, lactose was sterilely added to hot agar with YNB (~65 °C). Thanks to this, it was possible to obtain a final lactose concentration of up to 40%. Evaluation of yeast growth on the plates showed that over the entire range of lactose concentration tested, *K. marxianus* WUT240 showed good tolerance, and the calculated log(cfu/mL) did not show any significant differences; was estimated between 8.19 ± 0.20 and 8.77 ± 0.05 (Figure 5).

Taking into account the resistance of strain WUT240 to ethanol and lactose, fermentation was conducted in 20% WP (containing 140 g/L lactose). Because *K. marxianus* is a facultative anaerobe-respiratory yeast, two variants were tested, classic stationary and with gentle shaking cultures. During the first stage of the study, it was also tested whether the yeast biomass multiplied in Bioprocess I would still be active. After intensive production of 2-PE, yeast cells can be stressed, as 2-PE in concentrations of about 2 g/L is toxic. The results summarised in Table 3 showed that:

- yeast biomass is metabolically active and can be directly used for alcoholic fermentation;
- the gentle shaking does not significantly affect the final ethanol titer;
- yeast biomass originating from two-phase cultures generally allows obtaining higher ethanol titer and Y_P/X_ values than from control cultures.

The average ethanol titer during shaking cultures was 72 g/L after 72 h and was only 5% higher than the mean value for traditional stationary cultures. The calculated Y_PS_ was in the range of 0.47–0.53 g/g and was close to a maximum theoretical value of 0.54 g/g [32]. Interestingly, obtained ethanol concentrations exceeded the growth inhibitory value determined in the previous experiment. This phenomenon might imply that the *K. marxianus* WUT240 strain is more resistant to ethanol produced than to exogenously added one. Additionally, in the post-fermentation liquid glycerol (around 7–8 g/L) was also identified. Glycerol is an essential raw material used in important chemicals, food, and explosives applications [33]. It is also classified as one of the most promising compounds among the twelve biobased chemicals by the US Department of Energy [34]. In this regard, the possibility of its production on dairy waste is an additional asset and an attractive direction for further research.

After confirming the feasibility of using yeast multiplied by Bioprocess I, we analysed the effect of the initial biomass concentration on the ethanol yield in the study’s next step. These experiments were crucial for process standardisation, i.e., determining the optimal initial biomass content necessary to achieve satisfying results. For this purpose, the yeast biomass (from two-phase cultures with rice oil as the second phase) was freeze-dried, and the obtained ‘powder’ was used as inoculum. Two variants were tested:

- direct use of freeze-dried yeast;
- utilisation of yeast preincubated in a SAB medium for 4 h at 30 °C.

Typically, the inoculum concentration does not exceed 10%; in this study, 0.5–4% inoculum content was tested. Due to the lack of a noticeable improvement in productivity and also taking into account the possible costs associated with the energy spent on stirring, further studies of the traditional stationary cultures were carried out.

**Figure 6:**
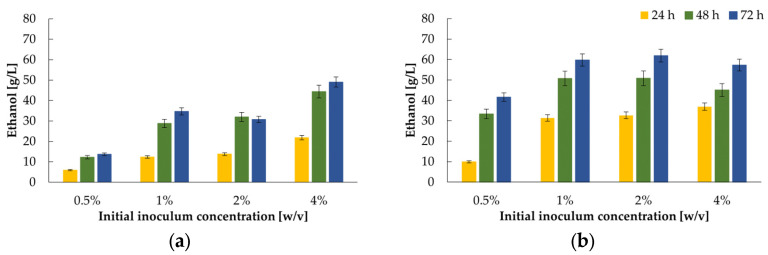
Ethanol production during 72 h batch cultures in 200WP medium with: (**a**) freeze-dried biomass; (**b**) preincubated for 4 h at 30 °C in SAB medium freeze-dried biomass of *K. marxianus* WUT240.

As shown in Figure 6a, a linear correlation between initial inoculum titer and ethanol concentration is generally observed when freeze-dried yeast biomass is directly used to start the fermentation process. The ethanol amount grew throughout the culture. The best results were obtained after 72 h and amounted to 49 g/L, 31 g/L, 35 g/L, and 14 g/L ethanol with 4%, 2%, 1%, and 0.5% initial inoculum concentration, respectively. For the most effective variant, the calculated Y_P/S_ was 0.35 g/g. Far better results were obtained with the initial revival of biomass (Figure 6b). As in the first system, the ethanol concentration increased during the course of the culture. Lower values were obtained with 0.5% inoculum (42 g/L ethanol), but for the following variants, similar results were estimated, around 60 g/L (1%), 62 g/L (2%), and 57 g/L (4%) ethanol, respectively. The yields corresponded to Y_P/S_ between 0.44 and 0.49 g/g.

**Figure 7:**
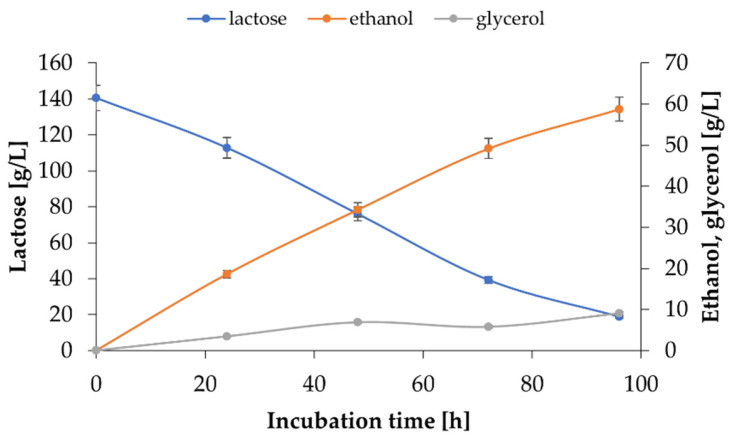
Changes in lactose, ethanol, and glycerol concentration during 96 h culture of *K. marxianus* WUT240 in WP200 medium at 30 °C in a 4.8-L bioreactor.

Ultimately, production on an enlarged scale was tested. 3 L stationary batch culture in a 4.8 L bioreactor with 1% preincubated inoculum of *K. marxianus* WUT240 was cultivated at 30 °C for 96 h (Figure 7). The 40-fold increase in culture volume did not affect the production itself, although it was necessary to prolong the incubation time to maximize fermentation yield. Due to incomplete substrate utilisation after 72 h around 49 g/L ethanol was formed, corresponding to a 20% decrease in the efficiency compared to production yield in Erlenmeyer flask scale cultures at the same time. Nevertheless, after 96 h, 59.14 ± 0.44 g/L ethanol was obtained, and 120.55 ± 0.44 0.70 g/L lactose was consumed, leading to process indicators, namely product per substrate and product per biomass yield coefficients, of Y_P/S_ = 0.48 g/g and Y_P/X_ = 3.11 g/g, respectively. 

### 2.4. Whey Permeate Bioconversion with K. marxianus WUT240 Leads to Its Organic Load Reduction

**Figure 8:**
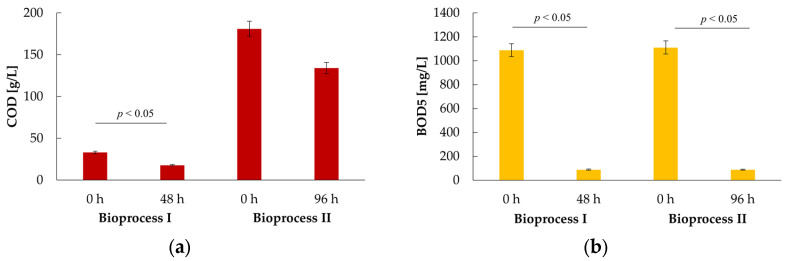
Whey permeate organic load reduction during 48 h cultures of *K. marxianus* WUT240 in 25WP (Bioprocess I) and 96 h cultures in 200 WP (Bioprocess II). The environmental impact of whey permeate was measured at the beginning (0 h) and the end of the cultures (of digestive liquids after main products extraction) as (**a**) COD and (**b**) BOD5 parameters.

The goal of our research was to determine whether efficient production of selected metabolites is possible and whether the proposed management of whey permeate will reduce its organic load indicators, namely COD and BOD5. For both bioprocesses, samples were taken at the beginning (before inoculation) and at the end of the cultures (Figure 8). 

For Bioprocess I, the estimated initial COD was 33 g/L and the final (after main products extraction) 17.7 g/L, corresponding to an R_COD_ = 46% (% COD reduction). In BOD5 analysis, an even higher decrease was noted, from 1088 mg/L to 88 mg/L (this results in relative BOD5 reduction R_BOD5_ = 92%). Organic load indicators analysis for Bioprocess II also proved a reduction in the harmful load of WP. It was similar under BOD5 (R_BOD5_ = 92%, decrease from 1110.2 mg/L to 87 mg/L), but for COD, it was only a 26% drop (from 181 g/L to 134 g/L) in comparison to Bioprocess I. 

## 3. Discussion

With a steady increase in the amount of waste generated from various industries, its valorisation becomes inevitable. In the present work, the possibility of whey permeate management was analysed. A non-conventional *Kluyveromyces marxianus* WUT240 strain was used in the study. Thanks to lactose permease and β-galactosidase activity, the strain can metabolise lactose, the main compound present in WP. This work aimed to develop a 2-step technology for 2-phenylethanol, bioferments, and ethanol production, coupled with the use of yeast biomass. 

With non-GMO, probiotic *K. marxianus* strain, we confirmed the possibility of using WP in a two-phase culture, leading to around 2 g/L pure 2-PE and fermented plant oils enriched with diverse VOCs (mainly ethyl acetate, 2-PE, 2-PEA, amyl alcohol and ethyl propionate). These compounds impart a pleasant sweet-floral aroma to the resulting bioferments and act as natural preservatives, thanks to their antimicrobial properties. A comparison of the VOCs distribution in samples from cultures on whey permeate and synthetic medium leads to the conclusion that the VOCs quantitative profile in *K. marxianus* WUT240 mostly depends on the carbon source used. To our knowledge, this is the first report showing two-phase cultures in WP with *K. marxianus*.

Thereafter, in the study, we also showed that multiplied in Bioprocess I yeast biomass is still metabolically active and can act as a biocatalyst in Bioprocess II of the designed technology. Notably, the study used a WP-based medium without any supplementation. Up to 75 g/L ethanol was obtained in an Erlenmeyer flask scale (with Y_P/S_ = 0.53 g/g, almost equal to a maximum theoretical value of 0.54 g/g). Subsequently, fermentation was tested in a 4.8-L bioreactor, and 59.14 ± 0.44 g/L ethanol was gained (Y_P/S_ = 0.48 g/g) after 96 h of incubation. The results presented here are competitive with the previously reported results. Gabardo et al. [35], using WP, produced 15–28 g/L ethanol with Y_P/S_ = 0.39–0.47 g/g with three different *K. marxianus* strains. With *K. fragilis* CECT 1123, Parrondo et al. [36] obtained 21.4 g/L ethanol at 34.1 °C (Y_P/S_ = 0.47 g/g). The ethanol concentration of 20 g/L was achieved after 140 h with *K. marxianus* DSMZ 7239 [37]. Interestingly, fermentation results in whey permeate presented in this paper are similar to or even better than described in whey-based media with different process modifications (e.g., yeast immobilisation, using cocultures, or fluidised bed bioreactors) [38,39,40,41]. 

One of the present study’s objectives was to verify whether the proposed technology reduces COD and BOD5 parameters. A significant decrease in these values was calculated (R_BOD5_ = 92%, R_COD_ = 26–46%); however, the estimates are still high enough to prevent the uncontrolled release of post-process water into the environment. Therefore, further development of downstream processing is necessary. It might be interesting to test the possibility of reusing the remaining wastewater in a later process. The final digest can, e.g., be examined as a substrate for methanisation, considering it a form of wastewater treatment and an additional energy gain.

## 4. Materials and Methods 

### 4.1. Yeast Strain and Media Composition

The yeast *Kluyveromyces marxianus* WUT240, deposited in a publicly available Warsaw University of Technology Yeast Collection (WUT YC) was used in the study. All data about the strain can be found on www.wutyeastcollection.pw.edu.pl (accessed on 19 January 2023) and in [13]. 

The composition of the media used in this study is listed in Table 4.

### 4.2. Effect of Exogenous Lactose, Ethanol and Ethyl Acetate on K. marxianus WUT240 Growth

To evaluate the potential inhibitory effect of lactose, ethanol, and ethyl acetate on the growth of *K. marxianus* WUT240, endurance tests were performed.

To examine the effect of lactose, overnight cultures of *K. marxianus* WUT240 were serially diluted 10-fold and spread in four repetitions on YNB-lactose agar plates containing YNB (6.7 g/L) (Biocorp, Warsaw, Poland) and lactose (Avantor Performance Materials Poland S. A., Gliwice, Poland) in a concentration between 20 and 400 g/L. Plates were incubated for 48 h, and afterward, differences in yeast growth were examined. 

To evaluate the effect of ethanol, overnight cultures of *K. marxianus* WUT240 were diluted in triplicate in 50 mL SAB medium (Biomaxima S. A., Lublin, Poland) to an optical density at 600 nm (OD600) of 0.15 and grown at 30 °C with shaking at 240 rpm for 48 h (LAB Companion SI-600R, Ramsey, MN, USA). When the cultures reached an OD600 value of approx. 0.6–0.8 ethanol (Avantor Performance Materials Poland S.A., Gliwice, Poland) was added to the cultures giving the final concentrations of 12.5–100 g/L. A culture without exogenous ethanol addition was conducted as a control.

### 4.3. Experimental Design

#### 4.3.1. 2-Phenylethanol Production in Two-Phase Batch Cultures in Shaking Flasks (Bioprocess I)

A sterile loop full of biomass from single colonies was used to inoculate the SAB medium in a 100 mL Erlenmeyer flask and grown overnight at 30 °C. The overnight culture was then diluted in 125 mL of WP25 medium in a 300 mL Erlenmeyer flask (Table 1), reaching an inoculum concentration of 2%. Batch cultures were incubated at 30 °C with shaking at 240 rpm (LAB Companion SI-600R, Ramsey, MN, USA) for 48 h in triplicate. When the cultures reached an OD600 value of approx. 0.8–1.0, one of the plant oils (rapeseed, linseed, or rice) (Oleofarm Sp. z o. o., Wroclaw, Poland) at a concentration of 20% (*v*/*v*) was added. As a control, culture without oil addition was conducted; 1 mL samples were collected at indicated time points to determine OD600, lactose, L-phe, and 2-PE concentration. At the end of the cultures, 10 mL samples were collected to determine the dry cell weight (DCW) and VOCs profile.

#### 4.3.2. Bioprocess I in 4.8-L Laboratory Bioreactor Scale

An overnight culture of the *K. marxianus* WUT240 strain in SAB medium was used to inoculate 2.4 L of WP25 medium in a 4.8-L bioreactor (Applikon EZ-control, Applikon Biotechnology, Schiedam, The Netherlands) equipped with pH, temperature, and dissolved oxygen control. The batch cultures were conducted for 48 h at 30 °C with a stirring speed of 250 or 500 rpm, and the airflow rate was maintained at 0.80 vvm (gas volume flow per unit of liquid volume per minute). When the cultures reached an OD600 value of approx. 0.8–1.0, rice oil in a concentration of 20% (*v*/*v*) was added.

During the cultures, 1 mL samples were collected in duplicate at indicated time points to determine OD600, lactose, L-phe, and 2-PE concentration. Additionally, at the end of the cultures, 10 mL samples were collected to determine the dry cell weight (DCW) and 100 mL samples for biochemical oxygen demand (BOD5) and chemical oxygen demand (COD) measurements.

#### 4.3.3. Fermentation of Lactose to Ethanol (Bioprocess II)

(I) For the determination of yeast biomass activity after Bioprocess I, the entire volume of two-phase cultures was centrifuged (5000× *g* for 5 min, 20 °C), the supernatant was separated, and the biomass was washed twice with sterile water. The biomass thus prepared was suspended in 150 mL of WP200 medium in 300 mL Erlenmeyer flasks and incubated at 30 °C, without or with gentle shaking (50 rpm), for 72 h.
(II) In order to determine the effect of initial biomass concentration on fermentation, the biomass multiplied in Bioprocess I was freeze-dried and subsequently weighed to obtain the selected inoculum concentration (0.5–4%, *w*/*v*). Two variants were tested, without and with prior revival (SAB medium, 30 °C, 240 rpm for 4 h) of lyophilised yeast. 75 mL cultures in 250 mL Erlenmeyer flasks were incubated stationary at 30 °C for 72 h.

Scale-up was also performed and cultures were carried out in a final volume of 3 L in a 4.8-L bioreactor. The process parameters were as follows: 1% inoculum, 200WP, 30 °C, 96 h.

During the cultures, 1 mL samples were collected at indicated time points to determine OD600, lactose, and ethanol concentration. At the end of the cultures, 10 mL samples were collected to determine the dry cell weight (DCW). For bioreactor scale tests after 96 h of incubation additionally 100 mL samples for biochemical oxygen demand (BOD5) and chemical oxygen demand (COD) measurements were collected.

### 4.4. COD and BOD5 Measurements

All the samples were centrifuged (5000× *g* for 5 min, 20 °C) to remove microbial cells and debris, and the COD and BOD5 were measured. The COD was determined using a NANOCOLOR COD 1500 kit (Machery-Nagel, Düren, Germany) according to ISO 1575:2002. BOD5 analyses were conducted with the Oxitop^®^-I SET2 system (Xylem Analytics Germany GmbH, Weilheim, Germany) and BOD seed inoculum (International Laboratory Supply, Ltd., Spring, TX, USA) according to manufacturer’s instructions. 

### 4.5. Analytical Methods

2-PE and L-phe concentration was determined by a high-performance liquid chromatography, HPLC (SYKAM chromatograph, Sykam GmbH, Eresing, Germany), with a DAD detector and a Bionacom Velocity C18-2 column (250 × 4.6 mm, 5 μm). An isocratic method comprising 70:30 2% formic acid/acetonitrile with 2% formic acid was used at a flow rate of 1 mL/min. A DAD detector was set at a wavelength of 256 nm. Lactose and ethanol content was determined by HPLC coupled with an RI detector and a SETREX IEX H+ column (300 × 8 mm column, Polymer IEX H form, 8 μm) under thermostatic control at 35 °C. The RI detector was also set at 35 °C to avoid fluctuations in detector responses. Samples were eluted isocratically using 9 mM H_2_SO_4_ as the mobile phase at a flow rate of 1 mL/min. 

Volatile Organic Compounds (VOCs) were determined in liquid samples of both water and oil phases obtained at the end of Bioprocess I according to [42].

To calculate the dry cell weight (DCW), 10 mL aliquots were centrifuged at 5000× *g* for 2 min; supernatants were transferred to clean tubes, and yeast pellets were dried at 85 °C until reaching constant weight. 

The cell density in liquid medium samples was monitored by measuring turbidity at 600 nm (OD600) using a VIS-7220G spectrophotometer (Beijing Rayleigh Analytical Instrument Corporation Co., Ltd., Beijing, China).

### 4.6. Calculations

The parameters used to evaluate the performance of the Bioprocess I and II included total 2-PE bioconversion, product yield coefficient, volumetric productivity, and theoretical conversion of L-phe to 2-PE and lactose to ethanol (Equations (1)–(6), respectively). The chemical and biochemical oxygen demand reduction were calculated as described in Equations (7) and (8), respectively. Colony forming units/mL in selected samples were calculated according to Equation (9).

(1) $$Total\;2PE\;concentration\;{2PE}_{tot}={(2PE}_{aq}*V_{aq}+{2PE}_{org}*V_{org})/V_{tot}(g/L)$$

(2) $$Product\;per\;substrate\;yield\;coefficient\;Y_{P/S}=\frac{P_{F}-P_{I}}{\left|S_{F}-S_{I}\right|}(g/g)$$

(3) $$Product\;per\;biomass\;yield\;coefficient\;Y_{P/X}=\frac{P_{F}-P_{I}}{\left|X_{F}-X_{I}\right|}(g/g)$$

(4) $$Volumetric\;productivity\;Q=\frac{P_{F}-P_{I}}{t}(mg/L/h)$$

(5) $$Theoretical\;maximum\;conversion\;of\;lactose\;to\;ethanol\;\eta_{1}=\left|S_{F}-S_{I}\right|*0.5368\left(g/L\right)$$

(6) $$Theoretical\;maximum\;conversion\;of\;Lphe\;to\;2PE\;\eta_{2}=\left|S_{F}-S_{I}\right|*0.7577\left(g/L\right)$$

(7) $$\%\;COD\;reduction\;R_{COD}=\frac{{COD}_{I}-{COD}_{F}}{{COD}_{I}}*100\;(\%)$$

(8) $$\%\;BOD5\;reduction\;R_{BOD5}=\frac{{BOD5}_{I}-{BOD5}_{F}}{{BOD5}_{I}}*100\;(\%)$$

(9) $$colony\;forming\;units\;cfu/mL=\frac{A*100}{d}$$

where: *2PE_aq_*—2-PE titer in water phase (g/L); *V_aq_*—volume of water phase (L); *2PE_org_*—2-PE titer in oil phase (g/L); *V_org_*—volume of water phase (L); *V_tot_*—total culture volume; *P_F_*—final product content (g); *P_I_*—initial product content (g); *S_F_*—final substrate content(g); *S_I_*—initial substrate content (g); *X_F_*—final biomass content (g); *X_I_*—initial biomass content (g); 0.5368—theoretical value of the coefficient of conversion of lactose to ethanol; 0.7577—theoretical value of the coefficient of conversion of L-phe to 2-PE; *COD_I_*—COD value for medium WP25 or WP200 (g/L), *COD_F_*—COD value at the end of Bioprocess I or II (g/L); *BOD*5*_I_*—BOD5 value for medium WP25 or WP200 (mg/L), *BOD*5*_F_*—BOD5 value at the end of Bioprocess I or II (mg/L); *A*—number of colonies; *d*—dilution sample, 100—conversion factor per mL. 

All analyses were performed between two to four repetitions and the data are presented as the mean ± standard deviation (SD). Statistical comparisons were performed between groups using Student unpaired *t*-tests; *p* < 0.05 was the criterion for statistical significance.

## 5. Conclusions

With a steady increase in waste generated from various industries, its valorisation becomes inevitable. This work aimed to develop a new technology for whey permeate management based on sequential bioprocesses toward the value-added product. Using 225 g/L whey permeate in total, we managed to produce 1.7–1.8 g/L pure 2-PE, 75 g/L ethanol, 8 g/L glycerol, and 160 mL/L of selected bioferments enriched in diverse VOCs. Moreover, multiplied yeast biomass (10–20 g/L) can serve as inoculum for subsequent processes or for preparing yeast extracts. The resulting biocompounds have also strong application potential for food, feed, cosmetic, chemical or energetic industries.

## Figures and Tables

**Table 1:** Parameters of 48 h two-phase batch cultures of *K. marxianus* WUT240 in WP25 medium at 30 °C. Analyses were performed in triplicate and data are presented as the mean ± SD.

Parameter	Variant Tested			
	Control	Rice Oil	Linseed Oil	Rapeseed Oil
OD600	6.21 ± 1.23	12.10 ± 0.15	11.50 ± 0.75	9.66 ± 0.27
DCW [g/L]	4.62 ± 0.46	6.43 ± 0.53	8.08 ± 0.18	7.79 ± 0.75
Lactose [g/L]	nd *	nd	nd	nd
L-phe [g/L]	1.37 ± 0.17	0.99 ± 0.09	0.98 ± 0.03	0.96 ± 0.03
2-PE_aq_ [g/L]	2.45 ± 0.17	1.82 ± 0.07	1.71 ± 0.01	1.73 ± 0.05
2-PE_org_ [g/L]	-	4.36 ± 0.01	4.10 ± 0.03	4.43 ± 0.18
2-PE_tot_ [g/L]	2.45 ± 0.17	2.33 ± 0.04	2.19 ± 0.02	2.27 ± 0.11
Q [mg/L/h]	51.04 ± 3.54	48.54 ± 0.83	45.63 ± 0.42	47.29 ± 2.29
Y_P/S_ [g/g]	0.67	0.58	0.54	0.56

* nd–not detected; DCW—dry cell weight [g/L]; Q—productivity [mg/L/h]; Y_P/S_—product per substrate yield coefficient [g/g].

**Table 2:** Parameters of 48 h two-phase batch cultures of *K. marxianus* WUT240 in WP25 medium at 30 °C in 4.8-L bioreactor. Analyses were performed in duplicate and data are presented as the mean ± SD.

Parameter	Variant Tested	
	250 rpm	500 rpm
DCW [g/L]	12.51 ± 2.72	11.35 ± 1.53
Lactose [g/L]	nd *	nd
L-phe [g/L]	1.25 ± 0.17	1.05 ± 0.09
2-PE_aq_ [g/L]	1.02 ± 0.17	1.44 ± 0.07
2-PE_org_ [g/L]	2.84 ± 0.13	3.73 ± 0.01
2-PE_tot_ [g/L]	1.38 ± 0.03	1.90 ± 0.04
Ethanol [g/L]	3.62 ± 0.45	nd
Q [mg/L/h]	28.83 ± 0.65	39.54 ± 0.42
Y_P/S_ [g/g]	0.37	0.48

* nd–not detected; DCW—dry cell weight [g/L]; Q—productivity [mg/L/h]; Y_P/S_—product per substrate yield coefficient [g/g].

**Table 3:** Parameters of 72 h batch cultures of *K. marxianus* WUT240 in WP200 medium at 30 °C. Analyses were performed in duplicate and data are presented as the mean ± SD.

Parameter	Variant Tested			
	I	II	III	IV
	Shaking cultures (50 rpm)			
OD600	10.48 ± 0.52	8.82 ± 0.44	9.50 ± 0.48	9.07 ± 0.45
DCW [g/L]	13.83 ± 0.69	16.71 ± 0.84	21.17 ± 1.26	19.64 ± 2.96
Lactose [g/L]	nd *	nd	nd	nd
Ethanol [g/L]	69.63 ± 3.48	70.51 ± 3.53	73.52 ± 2.68	74.69 ± 0.87
Y_P/X_ [g/g]	5.03	4.22	3.47	3.80
Y_P/S_ [g/g]	0.50	0.50	0.53	0.53
Glycerol [g/L]	7.65 ± 0.65	8.08 ± 0.40	7.75 ± 0.49	7.65 ± 0.38
	Stationary cultures			
OD600	8.75 ± 0.86	8.83 ± 0.44	8.92 ± 0.48	9.33 ± 0.59
DCW [g/L]	17.33 ± 0.87	15.03 ± 1.77	16.41 ± 1.32	19.33 ± 1.63
Lactose [g/L]	nd	nd	nd	nd
Ethanol [g/L]	68.92 ± 3.45	71.77 ± 1.59	68.82 ± 2.44	65.40 ± 3.27
Y_P/X_ [g/g]	3.98	4.78	4.19	3.38
Y_P/S_ [g/g]	0.49	0.51	0.49	0.47
Glycerol [g/L]	7.62 ± 0.38	7.37 ± 0.37	7.71 ± 0.39	6.85 ± 0.34

* nd—not detected; DCW—dry cell weight [g/L]; Y_P/X_—product per biomass yield coefficient [g/g]; Y_P/S_–product per substrate yield coefficient [g/g].Variants I–IV used yeast biomass multiplied in Bioprocess I, respectively: I—control culture; II—two-phase culture with rice oil; III—two-phase culture with linseed oil; IV—two-phase culture with rapeseed oil.

**Table 4:** Media composition.

Medium	Composition
SAB	SAB 5 g/L meat peptone, 5 g/L casein peptone, 20 g/L glucose (Merck, Darmstadt, Germany), pH 5.6
WP25	25 g/L whey permeate (Mlekpol, Grajewo, Poland), 5 g/L L-phe (Merck, Darmstadt, Germany), tap water, pH 5.4
WP200	200 g/L whey permeate, tap water, pH 5.1

## Data Availability

Data will be made available on request.

## Supplementary Materials

The following supporting information can be downloaded at: https://www.mdpi.com/article/10.3390/ijms24087560/s1. Reference [43] are cited in the supplementary materials.
