# Supplementary material for: Valorisation of Whey Permeate in Sequential Bioprocesses towards Value-Added Products–Optimisation of Biphasic and Classical Batch Cultures of Kluyveromyces marxianus
Source: Int J Mol Sci. 2023 Apr 20;24(8):7560. doi: 10.3390/ijms24087560 (PMC10146618; doi:10.3390/ijms24087560)
Supplement: Supplementary file 1 [file ijms-24-07560-s001.zip › ijms-2295538-supplementary.pdf]

International Journal of *Molecular Sciences*

MDPI

Supplementary File

Whey permeate valorization: from process optimization with non-conventional *Kluyveromyces marxianus* WUT240 strain to organic load reduction

Karolina Dreżek [1,*], Maria Sobczyk [1], Zoltán Kállai[2], Anna Detman[3] and Jolanta Mierzejewska [1]

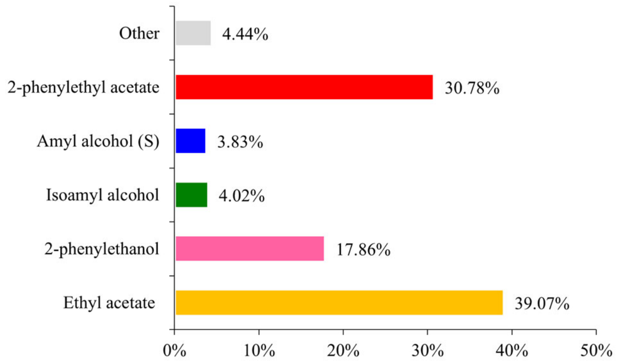

(a)

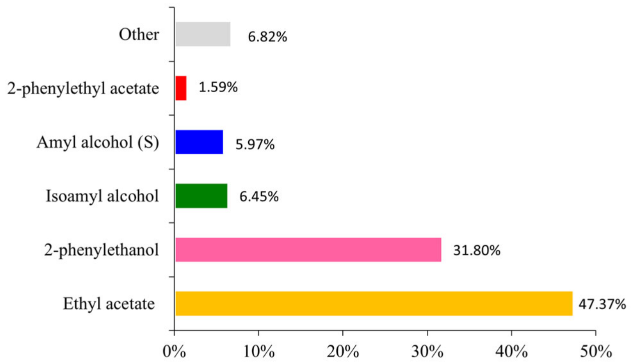

(b)

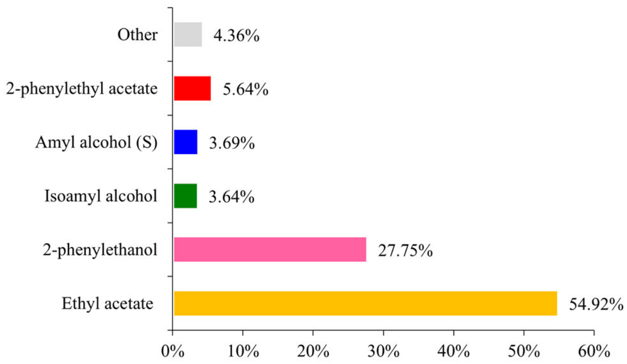

(c)

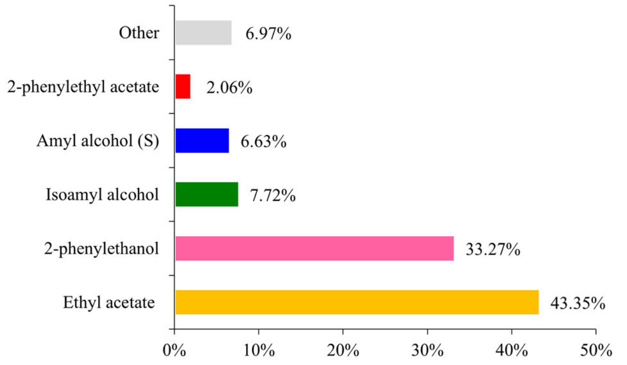

(d)

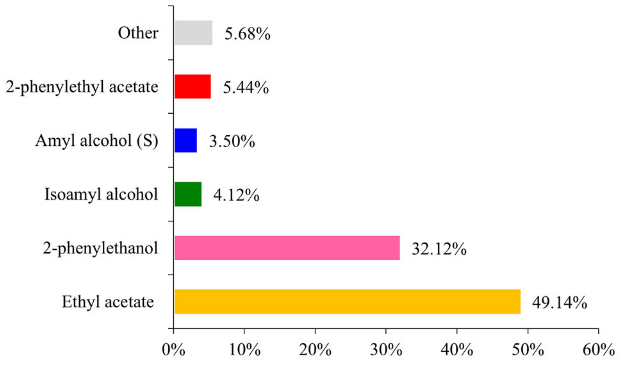

(e)

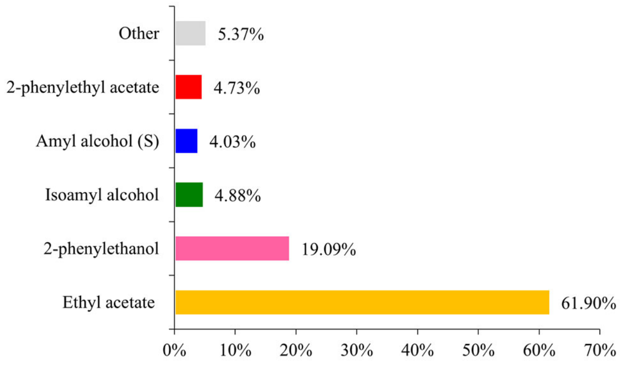

(f)

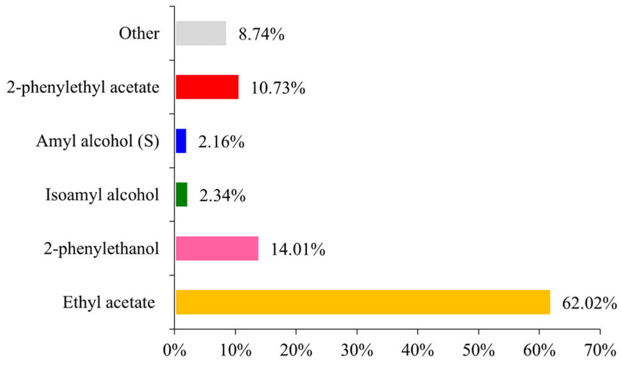

(g)

Supplementary Figure 1. VOCs profile in aqueous and organic samples obtained in *K. marxianus* WUT240 cultures in P8 medium incubated at 30°C, 240 rpm for 48 h: (**a**) control culture; (**b, d, f**) aqueous phase profile in two-phase cultures with rice oil, rapeseed oil and linseed oil, respectively; (**c, e, g**) organic phase profile in two-phase cultures with rice oil, rapeseed oil and linseed oil, respectively.

Composition of P8 medium can be found in Chreptowicz, K.; Wielechowska, M.; Główczyk-Zubek, J.; Rybak, E.; Mierzejewska, J. Production of Natural 2-Phenylethanol: From Biotransformation to Purified Product. *Food Bioprod. Process.* **2016**, *100*, 275–281, doi:10.1016/j.fbp.2016.07.011.
